# Case Report: Simultaneously diagnosed gastric adenocarcinoma and pernicious anemia – a classic association

**DOI:** 10.12688/f1000research.24353.2

**Published:** 2020-12-02

**Authors:** Syed Kamran, Mattias K. Dilling, Nathaniel A. Parker, Joel Alderson, Nathan D. Tofteland, Quoc V. Truong

**Affiliations:** 1 Department of Internal Medicine, University of Kansas School of Medicine, Wichita, KS, 67214, USA; 2 Department of Osteopathic Medicine, Kansas City University of Medicine and Biosciences, Kansas City, MO, 64106, USA; 3 Pathology Department, Ascension Via Christi St. Francis Hospital, Wichita, KS, 67214, USA; 4 Cancer Center of Kansas, Wichita, KS, 67214, USA

**Keywords:** Autoimmune gastritis, Parietal cells, Stomach cancer, Gastric adenocarcinoma, Pernicious anemia

## Abstract

Primary gastric cancer remains one of the most prevalent malignancies worldwide. Often patients remain asymptomatic until it is detected at an advanced stage with a poor prognosis. Thus, it’s characteristically difficult to initially diagnose until it becomes late stage, at which point prognosis becomes poor. Pernicious anemia is a classic risk factor for the development of primary gastric cancer, but is uncommonly seen in clinical practice. Over time, patients who produce the autoantibodies to intrinsic factor that cause pernicious anemia typically will present initially with clinically significant megaloblastic anemia and peripheral neuropathy. However, patients can also present with more nonspecific signs and symptoms. Thus, clinicians should remain vigilant as circulating anti-intrinsic factor antibodies only worsen the disease over time and increase the risk of developing primary gastric cancer. This report not only presents the rare concurrent diagnosis of pernicious anemia and gastric cancer, but also aims to increase clinical awareness of these two conditions’ classic association because early diagnosis and treatment significantly impacts morbidity and mortality.

## Introduction

Despite the decline in gastric cancer incidence rates over the past several decades, it remains one of the most common and fatal malignancies worldwide. Yearly, over one million cases are diagnosed with an estimated 780,000 annual mortality incidence ^1^. The high mortality of gastric cancer is in part attributed to late initial diagnosis ^2^. The diagnosis of gastric carcinoma often is delayed because up to 80% of patients are asymptomatic during the early stages of stomach cancer ^3^. Weight loss, abdominal pain, nausea and vomiting, early satiety, and esophageal reflux-like symptoms are often late signs of tumor progression ^4^. By the time many symptoms develop, the disease is almost invariably too far advanced for curative procedures ^2^. Classic physical exam findings, such as organomegaly (e.g. stomach, liver) or regional lymphadenopathy (e.g. Virchow’s node, Sister Mary Joseph’s nodule) should raise suspicions for a gastric malignancy ^3^. More common risk factors for development of gastric cancer include Helicobacter pylori infection, tobacco smoking, heavy alcohol use, age, diet, and non-Caucasian ethnicity ^3, 4^.

Although less common, pernicious anemia (PA) remains a classic risk factor for primary gastric cancer. The condition is defined as autoimmune destruction of the intrinsic factor (IF) glycoprotein, or destruction of the gastric body and fundal parietal cells that produce IF ^5^. Since IF plays a crucial role in the transportation and absorption of vitamin B12, the product of this deleterious autoimmune process is the characteristic megaloblastic anemia ^6^. Importantly, PA represents 20% – 50% of the adult cases of vitamin B12 deficiency worldwide ^7^.

PA often has an insidious onset, which can make early diagnosis difficult. Clinically, patients commonly present initially with nonspecific symptoms such as fatigue, weakness, and mild paresthesias ^7^. However, unintentional weight loss can occur in 50% of patients ^5^. Chronically severe vitamin B12 deficiency can lead not only lead to worse neurologic dysfunctions, but also glossitis and gastrointestinal issues ^6^. Clinically significant vitamin B12 deficiency manifests as neurologic dysfunction in 74% of patients ^8^. The classic late-stage neurologic complication of vitamin B12 deficiency is subacute combined degeneration of the posterior and lateral columns of the spinal cord due to demyelination. However, peripheral neuropathy and non-neurologic manifestations are more common ^5^.

Individuals with PA have long been suggested to be at increased risk for gastric cancer ^9^. The pathogenesis of gastric cancer arising from PA is thought to be due chronic inflammation with extensive atrophy of the gastric mucosa, leading to increased risk of progression to gastric neoplastic lesions ^10^. This report presents the uncommon, but classic association, of PA with primary gastric cancer. When patients present with abdominal symptoms, unintentional weight loss, megaloblastic anemia, and low vitamin B12 levels, we should consider the presence of PA and its associated complication of primary gastric cancer.

## Case report

A 61-year-old Hispanic female retail worker, for whom the only pertinent past medical history was intermittent social alcohol consumption, presented to the emergency department with epigastric pain. Symptom onset began three weeks prior to her initial presentation and had been progressively worsening. Her chief complaints were associated with heartburn, decreased appetite, weakness, and 60-pound unintentional weight loss over the past six months. The patient attributed all of the symptoms to her physically and mentally demanding occupation. She denied a personal history of nausea, vomiting, dysphagia, hematemesis, hematochezia, melena, or screening colonoscopy. Her family history was negative for any gastrointestinal diseases or malignancies.

**Figure 1.**
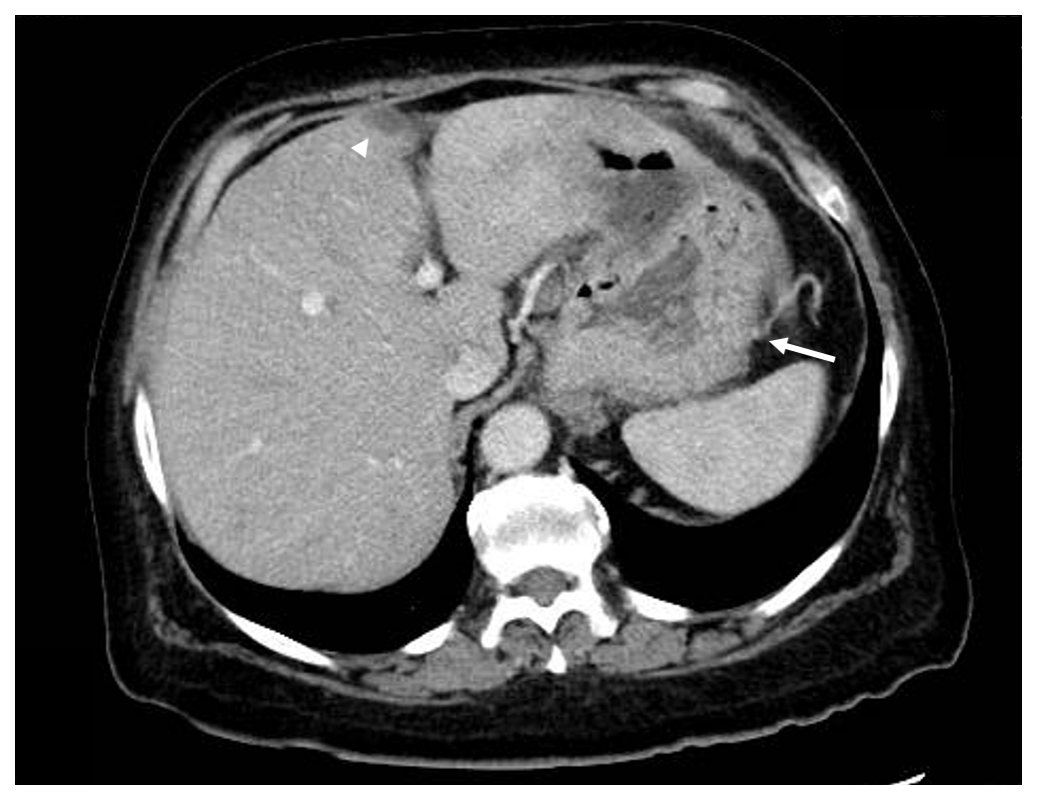
Abdominopelvic CT scan with contrast is primarily nonrevealing for a malignant process. Nonspecific gastric fold thickening in the fundus is observed ( *arrow*). An incidental finding in the liver was noted by a small focal hypoattenuation in the middle segment of the left lobe of the liver adjacent to the fissure for ligamentum teres ( *arrowhead*). This nodule was confirmed as PET-negative on later PET/CT studies.

**Figure 2.**
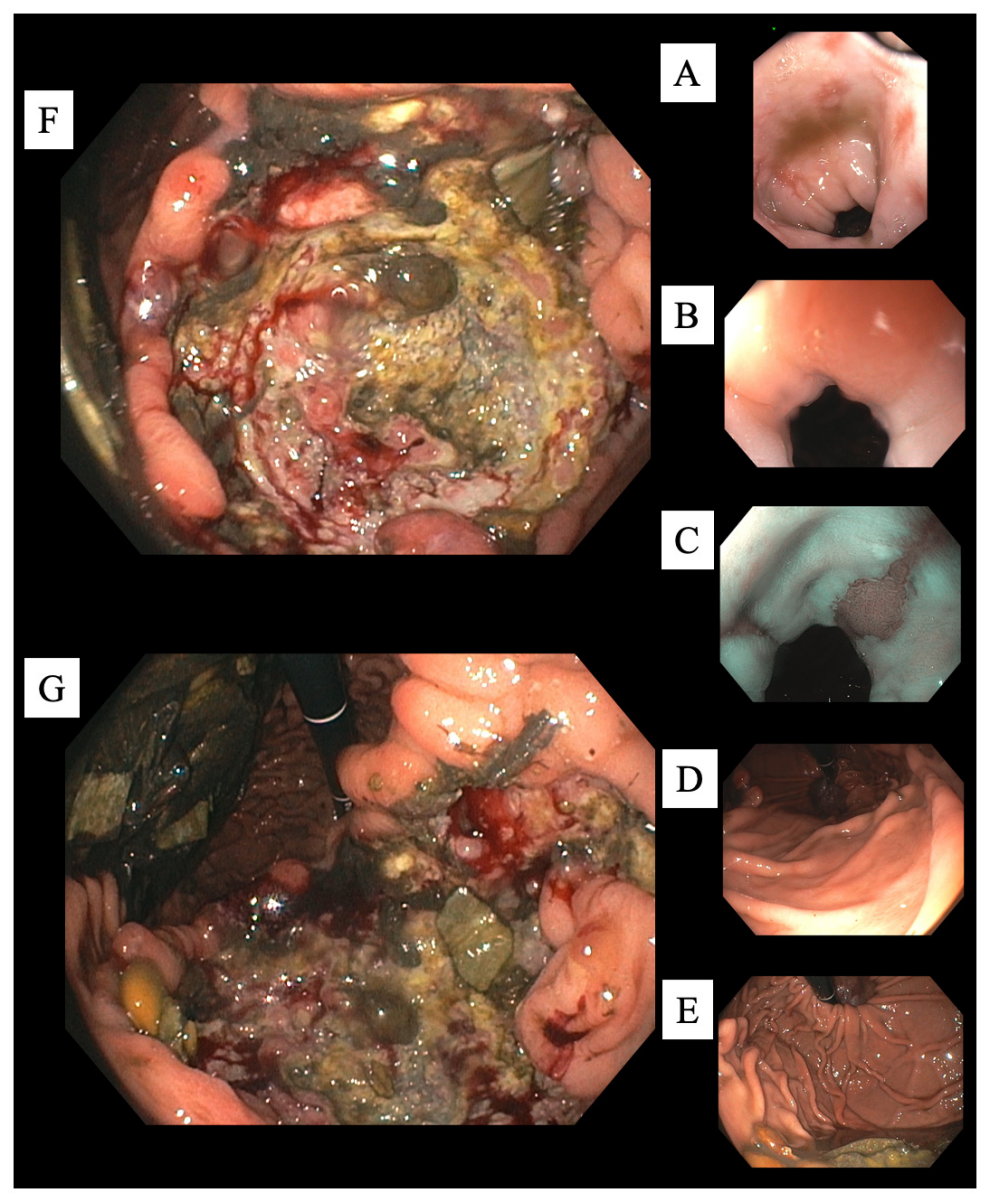
EGD demonstrates esophagitis and an extensive ulcer involving the entire lesser curvature of the stomach. (From proximal to distal.) ( **A**) Esophagitis. ( **B**) Gastro-esophageal junction. ( **C**) More esophagitis, and a tongue of columnar mucosa. ( **D**) Normal gastric cardia. ( **E**) Normal gastric fundus. ( **F**) Cavernous ulcer along the incisura (lesser curvature) with debris, food particles, and some central exudates ( **G**) Continued ulcer description. At the 12 o'clock position the scope is originating from the gastric cardia and fundus region. At 3 o'clock is the expected location of the pylorus. However, due to size and extent of the ulcer typical anatomy and landmarks were considerably distorted making visualization of the fundus from that particular EGD position not possible. At 6 o'clock the ulcer is shown to extend along the incisura. At 9 o'clock food debris is seen along the greater curvature.

Vital signs and measurements were unremarkable. A detailed physical examination was nonrevealing. Serum laboratory analysis was notable for a significant anemia, as well as a low vitamin B12 and iron deficiency ( Table 1). She was urgently managed with a restrictive transfusion strategy by one unit of leukoreduced packed red blood cells, which improved her symptoms and hemoglobin. Abdominopelvic imaging was obtained by computerized tomography (CT) scans with contrast, which were primarily equivocal ( Figure 1). Subsequently, she underwent esophagogastroduodenoscopy (EGD) for further evaluation. Esophageal findings included moderate esophagitis and salmon-colored mucosa at the gastroesophageal junction suggestive of Barrett's esophagus. Beyond the esophagus, a large 7-cm hiatal hernia was evident. An extensive, deep ulcer was noted to involve the entirety of the incisura and pre-pyloric area, as well as extended along the lesser curvature ( Figure 2). Multiple biopsies of the ulcer were obtained to evaluate for malignancy, as well as random gastric biopsies to evaluate for Helicobacter pylori colonization. *Helicobacter pylori* immunohistochemical (IHC) stain was ultimately negative. Due to the tumor's size, gross appearance, ulcerations, and bleeding erosions noted on EGD a malignant process was suspected. Based on the suspicions for an underlying malignant gastric neoplasm and the presence of a vitamin B12 deficiency, serum serologic testing for PA was obtained. Laboratory testing was positive for anti-IF confirming the diagnosis of PA ( Table 1).

**Table 1.** Biochemical analysis reveals an anemia. Corrected reticulocyte count and reticulocyte index for her age and gender suggests an inappropriate marrow response (reticulocyte index < 2) likely relating to the patient’s nutritional deficiencies, instead of bone marrow abnormalities. The low vitamin B12 level supports the lack of sufficient vitamin B12 in the blood. Serum IF-blocking antibody testing was not obtained on admission, but after EGD, which was positive and confirmed the diagnosis PA.

Laboratory findings	Result	Reference range
White blood cells	10.7	4.8 – 10.8 10 ^3^/µL
Hemoglobin	6.8	12 – 16 g/dL
Hematocrit	22.3	37 – 47 %
Mean corpuscular volume	81.1	82 – 99 fL
Red blood cell distribution width	15.7	11.5 – 14.5 %
Platelets	340	150 – 400 10 ^3^/µL
Reticulocyte count	1.9	0.6 – 2.5 %
Total iron concentration	8	50 – 17 µg/dL
Total iron binding capacity	299	286 – 569 µg/dL
Iron saturation	3	11 – 46 %
Transferrin	201	192 – 382 mg/dL
Ferritin	11	11 – 307 ng/mL
Vitamin B12	141	213 – 816 pg/mL
Folate	8.6	7 – 31.4 ng/mL
Hemoglobin A1c	5.6	4.1 – 5.6 %
Total protein	6.4	6.1 – 7.9 g/dL
Lactate dehydrogenase	99	98 – 192 U/L
Thyroid stimulating hormone	2.78	0.35 – 5.5 µIU/mL
Ethanol level	-	-
Fecal occult blood test	+	-
IF-blocking antibody	+	-
Parietal cell IgG	-	-

**Figure 3.**
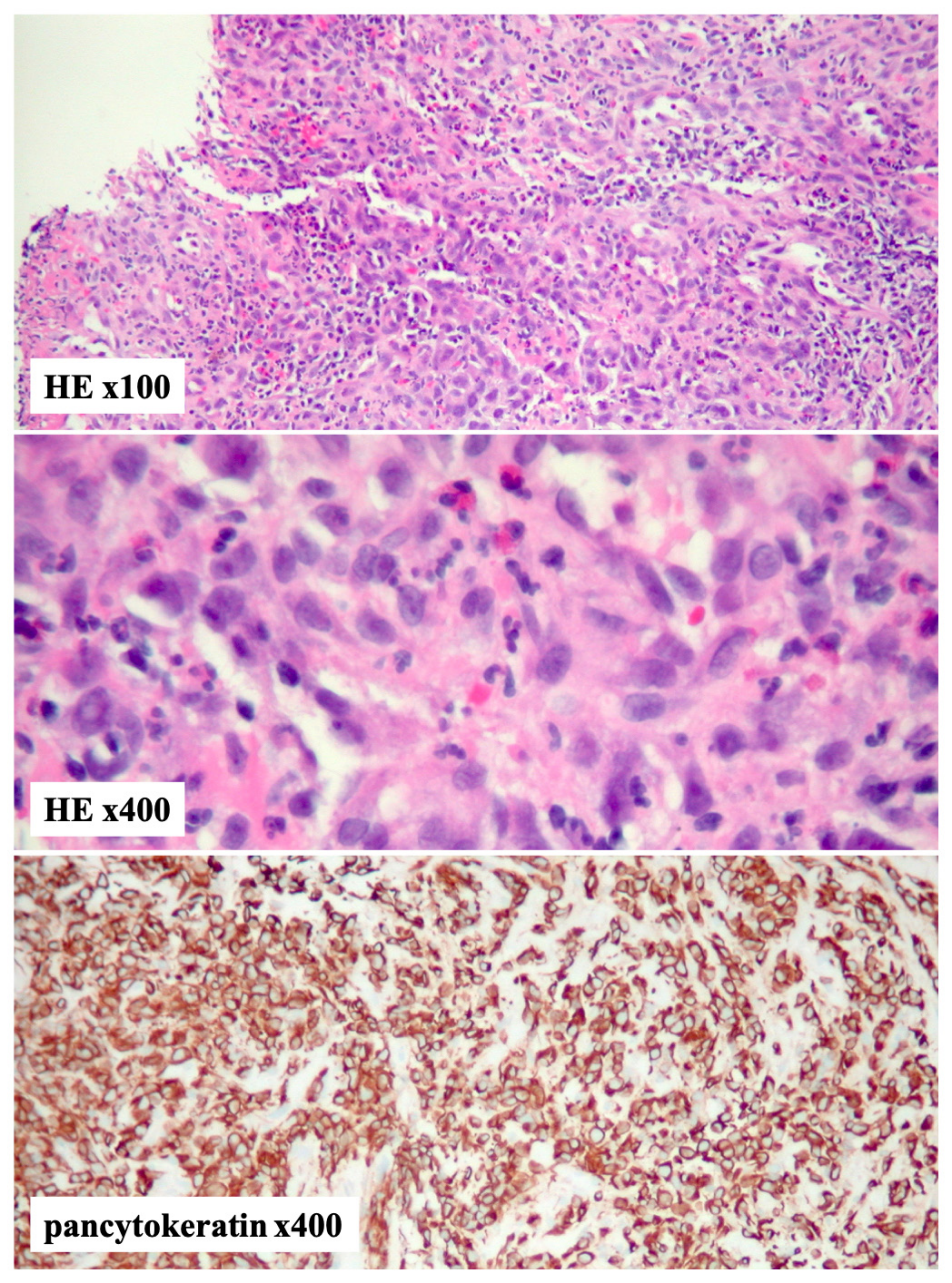
Pathology demonstrates an invasive poorly differentiated adenocarcinoma.

**Figure 4.**
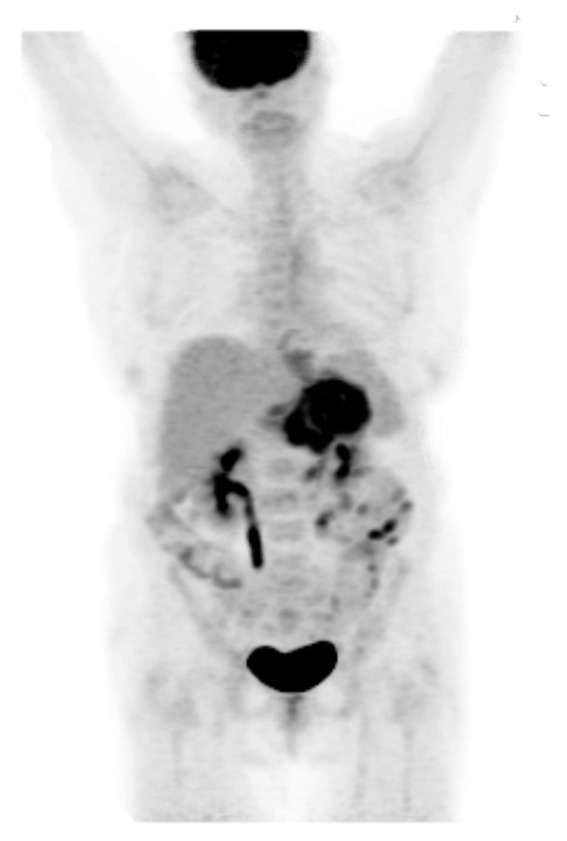
Pre-chemotherapy staging PET scan shows a locally advanced gastric cancer. PET from skull to mid-thigh reveals extensive, diffuse hypermetabolism throughout the gastric wall compatible with a PET-avid infiltrating gastric neoplasm. Imaging revealed involvement of at least one hepatogastric lymph node. Thus, the patient was determined to have stage III disease (T3N1). Scattered areas of contrast uptake within the bowel are likely physiologic and limit evaluation for lesions. Contrast uptake within the brain and genitourinary system are physiologic.

**Figure 5.**
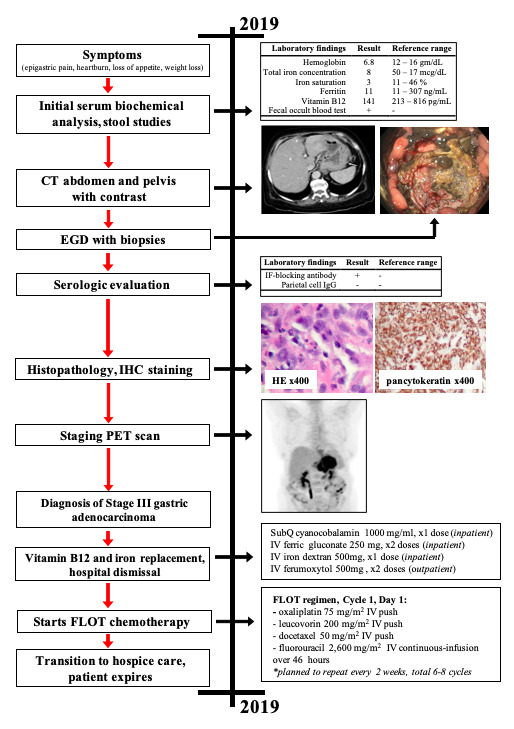
Case report timeline. Presented according to CARE guidelines.

Microscopically, antral mucosa demonstrated mild chronic gastritis. Histopathology revealed a diffuse-type, invasive poorly differentiated adenocarcinoma. IHC staining was only positive for pancytokeratin. The malignant-appearing cells lacked immunoreactivity for synaptophysin, CD45, and HER2 ( Figure 3). Together with histopathology and this IHC profile gastric adenocarcinoma was confirmed. She underwent a positron emission tomography (PET) scan for staging. The PET scan showed a localized but advanced gastric neoplasm associated with regional PET-avid lymphadenopathy. ( Figure 4). Thus, she was diagnosed with Stage II gastric adenocarcinoma ( Figure 5).

She was started on intravenous vitamin B12 and iron replacement (dose given in Figure 5). Her remaining symptoms improved slowly while she observed in post-operative period with twice-daily oral 40 mg pantoprazole and daily polyethylene glycol. She continued to improve following intravenous vitamin B12 and iron replacement and transitioned to a scheduled oral cyanocobalamin 1000 µg daily regimen. Oral iron supplementation was discouraged due to the propensity of oral iron medication to be significantly irritative to the gastric mucosal lining. She was dismissed from the hospital and established care with a local oncologist. Due to the nature of her disease, chemotherapy was recommended. The patient initiated treatment with the FLOT regimen (oxaliplatin, leucovorin, docetaxel, fluorouracil) for her advanced, invasive poorly differentiated gastric adenocarcinoma ( Figure 5). However, the patient did not tolerate chemotherapy well and only was able to endure one cycle. She wished to stop all therapies and was transitioned to hospice care. Two weeks after stopping chemotherapy, the patient expired.

## Discussion

The prevalence of PA is 0.1% in the general population, and approximately 2% in patients older than 60 years of age ^7^. The highest prevalence is seen in Northern Europeans, specifically those in Scandinavia and the United Kingdom ^11^. Several autoimmune diseases, such as type 1 diabetes mellitus, vitiligo, and autoimmune thyroid disease, have all been reported to have an increased association with the development of PA ^7^. The true prevalence of PA remains controversial. Assays to assess the vitamin B12 status of patients have historically been unreliable. Consequently, attempts to estimate the prevalence of vitamin B12 deficiency among the general population remains difficult ^12^. In addition, controversy remains regarding what level of vitamin B12 constitutes a deficiency. Even more problematic for patients and healthcare providers is a sub-clinical vitamin B12 deficiency. This can lead to patients returning frequently to the office over many years, presenting with a myriad of symptoms, and increasing their healthcare costs. Thirty percent of patients with PA the estimated time between symptom onset and diagnosis is 2 – 5 years. Fourteen percent of patients wait over ten years for a diagnosis. Thus, for those with equivocal, sub-clinically low vitamin B12 levels, or who present with symptoms discordant with their serum vitamin B12 levels, holotranscobalamin, the active form of vitamin B12, and methylmalonic acid testing can be considered ^12^.

 A diagnosis of PA is made when patients have a low vitamin B12 level and are positive for anti-IF antibody or anti-parietal cell antibody, or low vitamin B12 level in the presence of atrophic gastric mucosa seen on histopathology ^11^. PA is often clinically diagnosed prior to any subsequent development of cancer. Concurrent diagnosis of PA and gastric cancer, as with our patient, is exceedingly rare and often observed in patients who present late in the course of their disease.

Having a clinical awareness to the presence of a vitamin B12 deficiency is often sufficient to begin the workup. However, reliance on biochemical evidence alone to suggest low vitamin B12 is not recommended. Various occult malignancies can falsely elevate vitamin B12. Thus, a vitamin B12 deficiency can be masked. Given the underlying pathophysiology of PA, patients with a known history of autoimmune conditions are at an increased risk to develop anti-IF autoantibodies. Anti-parietal cell antibodies are present in around 90% of patients with PA, but have low specificity. Conversely, anti-IF antibodies are found in around 60% of patients with PA, but are considered much more specific for the disease ^11^. Similar to most serologic tests, autoantibody testing for IF has a low sensitivity. In contrast, specificity remains high and the presence of anti-IF autoantibodies is diagnostic for PA ^13^.

Based on the American Society of Gastrointestinal Endoscopy, following the diagnosis of PA endoscopy, evaluation by EGD is recommended, especially if gastrointestinal symptoms are present ^14^. However, only about 25% of patients diagnosed with PA patients undergo subsequent upper endoscopic screening ^14^. If endoscopic findings are suspicious for a neoplastic process, tissue sampling can aid in the diagnosis of a gastric malignancy. Commonly, gastric specimens are immunoreactive to markers for carcinoma, such as pancytokeratin. However, the diagnosis can be challenging since primary gastric adenocarcinoma cells commonly exhibit partial synaptophysin immunoreactivity ^15^.

The mainstay treatment for PA involves parenteral replenishment. Due to its autoimmune nature, treatment is typically needed indefinitely. High-dose oral or sublingual vitamin B12 therapy can also be used, provided a good adherence to treatment and a positive response is seen ^16^. Intranasal formulations are not recommended due to the higher cost, side effects, and inconsistent absorption. Following definitive surgical management by gastrectomy, indefinite treatment with parenteral vitamin B12 is still appropriate ^16^.

Research into anti-IF autoantibodies has been ongoing as a possible immunotherapy target. While no immunotherapeutic agent currently exists, experts have proposed a specific clone of CD4+ T-cells to be involved in the destruction of the gastric cells in PA and autoimmune gastritis ^17^. The finding of these T-cells could lay the groundwork for developing new immunotherapies against the T-cells in future studies, especially in PA patients in whom the anti-IF autoantibodies are not present.

The relative risk for gastric adenocarcinoma in PA patients is as high as 6.8 (95% CI: 2.6–18.1) ^8^. After the initial diagnosis of PA, the risk of developing gastric cancer increases. The association between PA and gastric cancer becomes strongest 6 years after the first record of PA (OR: 2.53). In contrast, if PA is discovered and treated early, gastric cancer is less likely to develop (OR: 1.77) ^10^. Thus, clinicians should remain vigilant as unnoted circulating anti-IF autoantibodies worsen PA and increase the risk of patients developing gastric cancer overtime ^10, 12^.

Once gastric cancer develops and is ultimately diagnosed following histopathologic investigation, the prognosis often varies based on several factors. Overall, proximal tumors near the gastroesophageal junction and cardia have a poorer prognosis ^18^. Gastric cancer has a male predominance and a higher mortality rate in men ^1^. Compared to surgical intervention alone or concomitantly with chemotherapy, chemotherapy and radiation increases the chances of achieving complete remission. If the patient is willing and able to undergo surgical management, upper GI endoscopic resection or gastrectomy is recommended. Following surgical intervention there are currently no randomized trials to help guide posttreatment surveillance strategies ^19^. The National Comprehensive Cancer Network suggests a risk-stratified surveillance strategy that is tailored to individual patients. No specific guidance is provided, but factoring tumor staging and if a patient underwent endoscopy or gastrectomy is recommended ^20^. Vitamin B12 and iron must be closely monitored along with bone health, especially in female patients who have undergone a total gastrectomy. Follow-up PET/CT scans can be considered as they are indicated clinically ^21^.

## Conclusion

Primary gastric cancer remains one of the most prevalent malignancies worldwide and is associated with poor outcomes. This is likely due to patient’s remaining asymptomatic until late-stage progression. Thus, early detection is paramount. PA is uncommonly seen in clinical practice, but remains a classic risk factor for the development of primary gastric cancer.

Identifying pertinent physical exam features and pairing them with lab findings of a vitamin B12 deficiency can be crucial steps in uncovering the gastric cancer early. In severe cases such as this patient, it is important to obtain proper radiographic imaging and an EGD. Unfortunately, only a minority of patients diagnosed with PA undergo subsequent gastric cancer screening with upper endoscopy. Thus, there is a need for increased clinical awareness of these two conditions’ classic association.

## Data availability

All data underlying the results are available as part of the article and no additional source data are required.

## Consent

Written informed consent for publication of their clinical details and clinical images was obtained from the patient.
