# Supplementary material for: Case Report: Simultaneously diagnosed gastric adenocarcinoma and pernicious anemia – a classic association
Source: F1000Res. 2020 Dec 2;9:604. Originally published 2020 Jun 15. [Version 2] doi: 10.12688/f1000research.24353.2 (PMC7656275; doi:10.12688/f1000research.24353.2)
Supplement: Peer review history

# Peer review: Reviewer response for version 1

**DOI:** 10.5256/f1000research.26868.r74105

**Published:** 2020-11-10

**Authors:** Martyn Hooper MBE

**Affiliations:** 1 The Pernicious Anaemia Society, Bridgend, UK

The Pernicious Anaemia Society's helpline often receives calls from newly diagnosed patients who want to know how likely they are to develop stomach cancer. It is the third most common subject for calls.

 This paper is to be commended for addressing the issues surrounding Pernicious Anaemia and Stomach Cancer, however, the numbers and percentages given in relation to PA are disputable simply because the assays used to assess the B12 status of patients is so unreliable. Consequently any attempt to estimate how prevalent B12 Deficiency is amongst the general population is difficult to say the least. Then there's the problem of what levels constitutes a deficiency or, even more problematic, a sub-clinical deficiency. 

 This problem often leads to patients spending many years making return visits to primary care doctors and presenting with a wide range of symptoms - 30% of the PA Society waited between two and five years before being given an explanation of their worsening symptoms, with 14% waiting over ten years. Perhaps the 'Active B12 ' trust (holotranscobalamin) along with Homocysteine and Methylmalonic Acid might be considered if the patient's clinical picture is discordant with the serum B12 results.

 There is another problem worth mentioning - only around 40-50% of patients presenting with the symptoms of Pernicious Anaemia will have any enlarged red blood cells and many won't have any anaemia.

 If the patient is assessed as being deficient in B12 the assay to determine whether the deficiency is due to autoimmune pernicious anaemia is also seriously flawed - something addressed by the Guideline from the British Committee for Standards in Haematology referenced by the authors. With there being little chance of the return of the Schilling Test anytime soon clinicians should be aware of the failings of the competitive binding luminescence assay to diagnose PA.

 It's interesting to note that members of the Pernicious Anaemia Society report that 82% of members have some gastrointestinal problems. The most common is sudden unaccountable bouts of diarrhoea which patients treat using strong probiotics although it can take up to a month for the patient to find any relief from their symptoms.

 The Pernicious Anaemia Society is campaigning to have all newly diagnosed patients to automatically be referred for endoscopy and colonoscopy to detect any neuro-endocrine tumours which patients are particularly susceptible to.

Are enough details provided of any physical examination and diagnostic tests, treatment given and outcomes?

Yes

Is the case presented with sufficient detail to be useful for other practitioners?

Yes

Is sufficient discussion included of the importance of the findings and their relevance to future understanding of disease processes, diagnosis or treatment?

Yes

Is the background of the case’s history and progression described in sufficient detail?

Partly

Reviewer Expertise:

Founder and current Executive Chairman of the Pernicious Anaemia Society - reg. charity no. 1147839

I confirm that I have read this submission and believe that I have an appropriate level of expertise to confirm that it is of an acceptable scientific standard.

## References

[1] : Patient journeys: diagnosis and treatment of pernicious anaemia. *Br J Nurs*.23(7) : 10.12968/bjon.2014.23.7.376 376-81 10.12968/bjon.2014.23.7.376 24732991

# Peer review: Reviewer response for version 1

**DOI:** 10.5256/f1000research.26868.r67562

**Published:** 2020-07-27

**Authors:** Talha Badar

**Affiliations:** 1 Division of Hematology and Oncology, Medical College of Wisconsin, Milwaukee, WI, USA; 2 Mayo Clinic, Jacksonville, FL, USA

Kamran *et al.* presented a case report and literature review of gastric cancer with concurrent diagnosis of PA. My comments are below: 

1. Based on my assessment of this report, one could easily argue that this could be someone with history of GERD due to hiatal hernia leading to Barrett's esophagus leading to GE junction adenocarcinoma. The authors should focus on differential diagnosis of conditions leading to GE junction/gastric adenocarcinoma. 
2. Prior history of GERD? (Especially with barret esophagus findings, hiatal hernia), eating habits? Occupational and family history? All to be known in patients with gastric cancer to look for possible etiology for cancer diagnosis. 
3. Could it be a GE junction tumor, extending to stomach?
4. What is sensitivity and specificity of the IF antibody assay?
5. Why was FLOT chemotherapy used, why not upfront Sx for Stage 1b disease and adjuvant therapy based on surgical pathology? This should be explained in more detail. 
6. Figure 5 is good, I suggest to mention the timeline in weeks/days/months from presentation --> diagnosis --> treatment --> transition to hospice. 
7. Introduction, paragraph 3, the third sentence needs better structuring. 
8. The references need to be better formatted, especially 5, 13 and 19. 

Are enough details provided of any physical examination and diagnostic tests, treatment given and outcomes?

Partly

Is the case presented with sufficient detail to be useful for other practitioners?

Partly

Is sufficient discussion included of the importance of the findings and their relevance to future understanding of disease processes, diagnosis or treatment?

Yes

Is the background of the case’s history and progression described in sufficient detail?

Partly

Reviewer Expertise:

Malignant Hematology---> Leukemia/myeloid disorder and stem cell transplant/cellular therapy.

I confirm that I have read this submission and believe that I have an appropriate level of expertise to confirm that it is of an acceptable scientific standard, however I have significant reservations, as outlined above.

# Response

**Published:** 2020-07-30

**Authors:** Nathaniel Parker

**Affiliations:** Kansas University School of Medicine, USA

Based on my assessment of this report, one could easily argue that this could be someone with history of GERD due to hiatal hernia leading to Barrett's esophagus leading to GE junction adenocarcinoma. The authors should focus on differential diagnosis of conditions leading to GE junction/gastric adenocarcinoma. 

**- Comment acknowledged. This request is out of the scope for this article, and does not align with the article's purpose.**

Prior history of GERD? (Especially with barret esophagus findings, hiatal hernia), eating habits? Occupational and family history? All to be known in patients with gastric cancer to look for possible etiology for cancer diagnosis. 

**- All pertinent and/or available information has been presented and detailed in the case description section.**

Could it be a GE junction tumor, extending to stomach?

**- All pertinent and/or available information has been presented and detailed in the case description section. Also, this is unlikely based on the EGD images and summary of the report detailed in the article.**

What is sensitivity and specificity of the IF antibody assay?

**- Please see Description section, paragraph 3, sentence 6.**

Why was FLOT chemotherapy used, why not upfront Sx for Stage 1b disease and adjuvant therapy based on surgical pathology? This should be explained in more detail. 

**- All pertinent and/or available information has been presented and detailed in the case.**

Figure 5 is good, I suggest to mention the timeline in weeks/days/months from presentation --> diagnosis --> treatment --> transition to hospice. 

**- Comment acknowledged. The authors decline to make the suggested change based on the differences in figure style presentation not providing additional value and clarity to the overall figure and article.**

Introduction, paragraph 3, the third sentence needs better structuring. 

**- Comment acknowledged. The authors decline to make the suggested change. This sentence is adequate and its current structure is justified.**

The references need to be better formatted, especially 5, 13 and 19. 

**- Comment acknowledged. The authors decline to make the suggested change because the current format of this reference was made by the Editors.**

# Peer review: Reviewer response for version 1

**DOI:** 10.5256/f1000research.26868.r66908

**Published:** 2020-07-24

**Authors:** Faustina N A. Sackey, Ph.D., FMWI

**Affiliations:** 1 Loeb Health Research Institute at Ottawa Hospital, University of Ottawa, Ottawa, ON, Canada

The manuscript entitled “Case Report: Simultaneously diagnosed gastric adenocarcinoma and pernicious anemia – a classic association” presented a case about a 61-year-old woman who suffered both pernicious anemia and gastric cancer, and even after various treatments later succumb due to advancement of the disease. The manuscript was well-written with all the essential details regarding the appropriate tests leading to the patient’s diagnosis and treatment. However, I have the following concerns:

 1. Title:

- Please keep it simple. My suggestion: “Simultaneous diagnosis of gastric adenocarcinoma and pernicious anemia – a classic association.”

2. Abstract:

- It would be great to include the disease prevalence or mortality ranking since an Abstract is what readers would glance first and must represent the text. Once prevalence is mentioned, statistics must be indicated. There is GLOBOCAN 2018 statistics about the global ranking of prevalence and mortality to check and include.

3. Introduction:

- What is the median overall survival (OS) of gastric cancer since the disease is largely asymptomatic? Please check and add.

4. Discussion:

 I know the authors made mention of autoimmune destruction of the intrinsic factor (IF) glycoprotein leading to the perturbation of Vitamin D absorption. Is this the only pathogenesis of gastric cancer, or are there other possibilities? Please check and include so that readers would have the full picture of the etiology of the disease.

 Since the authors said part of their aims of the study is to increase clinical awareness of the classic association of PA and gastric cancer, due to the asymptomatic nature of gastric cancer, early diagnosis is quite challenging. Create one more paragraph in the Discussion to suggest remedies that would curtail identification of the disease in advance stages. Examples:

1. Cut-off age to perform an annual screening of individuals, as it is commonly done for colorectal cancer.
2. Family history of PA and gastric cancer should aid in identifying high-risk individuals and be screened for epidermal growth factor receptor (EGFR) overexpression, a potent biomarker for poor prognosis, and expressions of both vascular endothelial growth factor A (VEGFA), and mutations of the P53 tumor suppressor gene (TP53).
3. Screening high-risk individuals for the pathogen, H. pylori infections.

Lastly, it will be excellent for the authors to use their experience to come up with feasible and preferred treatment options like surgical resection performed as total or subtotal gastrectomy; if chemotherapy is to be used, what drugs? 5-FU/leucovorin?

 Targeted therapy: Ramucirumab, a monoclonal antibody against vascular endothelial growth factor receptors 2, anti-EGFR antibody, nimotuzumab, together with vitamin B12 therapy to resolve the anemia.

Are enough details provided of any physical examination and diagnostic tests, treatment given and outcomes?

Yes

Is the case presented with sufficient detail to be useful for other practitioners?

Yes

Is sufficient discussion included of the importance of the findings and their relevance to future understanding of disease processes, diagnosis or treatment?

Partly

Is the background of the case’s history and progression described in sufficient detail?

Yes

Reviewer Expertise:

Oncology, Nephrology, Developmental Heart Defects.

I confirm that I have read this submission and believe that I have an appropriate level of expertise to confirm that it is of an acceptable scientific standard.

## References

[1] : Epidemiology of gastric cancer: global trends, risk factors and prevention. *Prz Gastroenterol*.2019;14(1) : 10.5114/pg.2018.80001 26-38 10.5114/pg.2018.80001 30944675PMC6444111

[2] : Gastric cancer review. *J Carcinog*.2014;13: 10.4103/1477-3163.146506 14 10.4103/1477-3163.146506 25589897PMC4278094
